# High-throughput sorting of mosquito larvae for laboratory studies and for future vector control interventions

**DOI:** 10.1186/1475-2875-11-302

**Published:** 2012-08-28

**Authors:** Eric Marois, Christina Scali, Julien Soichot, Christine Kappler, Elena A Levashina, Flaminia Catteruccia

**Affiliations:** 1 Institut de Biologie Moléculaire et Cellulaire, INSERM U963, CNRS UPR9022, 15 rue René Descartes, 67084, Strasbourg, France; 2 Division of Cell and Molecular Biology, Imperial College London, Imperial College Road, London, SW7 2AZ, UK; 3 Department of Vector Biology, Max-Planck Institute for Infection Biology, Chariteplatz 1, 10117, Berlin, Germany; 4 Dipartimento di Medicina Sperimentale e Scienze Biochimiche, Università degli Studi di Perugia, Terni, 05100, Italy; 5 Department of Immunology and Infectious Diseases, Harvard School of Public Health, 665 Huntington Avenue, Boston, MA, 02115, USA

**Keywords:** Malaria, Mosquito, Transgenesis, Fluorescence-assisted sorting, Sexing, *Anopheles gambiae*, piggyBac

## Abstract

**Background:**

Mosquito transgenesis offers new promises for the genetic control of vector-borne infectious diseases such as malaria and dengue fever. Genetic control strategies require the release of large number of male mosquitoes into field populations, whether they are based on the use of sterile males (sterile insect technique, SIT) or on introducing genetic traits conferring refractoriness to disease transmission (population replacement). However, the current absence of high-throughput techniques for sorting different mosquito populations impairs the application of these control measures.

**Methods:**

A method was developed to generate large mosquito populations of the desired sex and genotype. This method combines flow cytometry and the use of *Anopheles gambiae* transgenic lines that differentially express fluorescent markers in males and females.

**Results:**

Fluorescence-assisted sorting allowed single-step isolation of homozygous transgenic mosquitoes from a mixed population. This method was also used to select wild-type males only with high efficiency and accuracy, a highly desirable tool for genetic control strategies where the release of transgenic individuals may be problematic. Importantly, sorted males showed normal mating ability compared to their unsorted brothers.

**Conclusions:**

The developed method will greatly facilitate both laboratory studies of mosquito vectorial capacity requiring high-throughput approaches and future field interventions in the fight against infectious disease vectors.

## Background

Vector-borne infectious diseases are a major scourge for humanity. Malaria alone, caused by *Plasmodium* parasites transmitted by the bite of infected female *Anopheles* mosquitoes, annually infects 250 million people worldwide and kills close to one million, mostly children in sub-Saharan Africa [1]. Past attempts at curbing the disease by the massive use of insecticides and of insecticide-impregnated bed nets have promoted the spread of genetic resistance to a wide range of insecticides across mosquito populations. This is reducing the impact of insecticide-based control methods, and novel approaches to control vector populations are urgently needed to roll back the disease. A similar challenge is posed by the control of *Aedes* mosquito species transmitting viral diseases including yellow fever, dengue and chikungunya.

Vector control methods that specifically target the desired species represent a valid and environmentally friendly alternative to insecticides. Dating from the 1950s, the sterile insect technique (SIT) is a species-specific control strategy that has been successfully used to reduce the population size of insect pests such as the screw worm and the Mediterranean fruit fly [2]. For mosquito SIT ([3]; reviewed in [4,5]), large numbers of male insects must be produced in breeding facilities, sterilized by γ-ray irradiation, and released in the field to compete with wild males for mating with wild females. The SIT approach is well suited for *Anopheles* mosquitoes, as the majority of females mate once in their lifetime and use the sperm stored in their spermatheca to fertilize egg batches produced every time they take a blood meal [6]. This implies that if a female copulates with a sterile male, she will not mate again and will lay only infertile eggs. Repeated releases of sterile males over a given area would, therefore, achieve a local drop in vector populations and a consequent decrease in malaria transmission. However, labour-intensive procedures for selection of male-only populations and a large variability in the efficiency of sterilization have to date posed vast blocks for a massive application of this approach.

Since the turn of the century, vast progress has been made in the generation of molecular and genetic tools for studies on *Anopheles* mosquitoes. The sequencing of the *Anopheles gambiae* genome [7] has allowed the identification of thousands of genes that shape the biology and behaviour of this main malaria vector. These genomic advances, combined with the development of transgenic technologies [8] to modify the mosquito genome and the possibility of silencing gene expression through RNAi [9], are facilitating studies on the biological processes that are crucial to the ability of *Anopheles* mosquitoes to transmit malaria. Importantly, they also offer a basis for novel vector control strategies to complement insecticide treatments. Among the advocated novel approaches are genetic control strategies related to SIT, such as the release of insects carrying a dominant lethal (RIDL), that are currently being developed for the dengue vector, *Aedes aegypti*[10,11]. In RIDL, released male mosquitoes carry a transgene that makes their female progeny unviable or infertile. Further alternative strategies propose to alter the genetic make-up of mosquito populations to reduce their vectorial capacity traits, for instance by rendering mosquitoes resistant to human pathogens [12-14]. The introgression of natural genetic traits or of synthetic transgenic constructs interfering with the mosquito vectorial capacity would lead to the replacement of natural disease-transmitting populations with mosquitoes that do not transmit infectious agents, a process that could be enhanced by artificial gene drive systems [15,16].

Regardless of the final goal of the control programme (population eradication through SIT/RIDL or population replacement), when targeting mosquito vectors male-only populations must be released, since females may (i) contribute to an unwanted increase of the mosquito populations if not sterile, (ii) mate with the released males thereby reducing the efficacy of the trial, and (iii) participate in disease transmission. This underlines the need for high-throughput sexing tools for mosquitoes: males carrying the desired sterility or disease-resistance trait need to be produced on an industrial scale to reach the vast numbers (millions) necessary for a release programme. It is essential that the released males, whether sterile or not, are fully competitive for mating with field females. Therefore, the mechanism utilized for inducing sterility/refractoriness, the mass rearing conditions and the sex sorting procedures must have no impact on overall male fitness.

Proof-of-principle evidence that automated separation of the sexes is achievable in *A. gambiae* mosquitoes was previously provided using a flow cytometry machine (COPAS®, Union Biometrica) to separate males from females based on the expression of a sperm-specific fluorescence marker in the testis [17]. The usefulness of this system was limited by the fact that male individuals could be identified only during late larval stages, at the onset of sperm development. Here, the full potential of this high-throughput system is achieved by the use of early sex-specific transgenic markers. This system allows the efficient and fully accurate separation of the desired phenotypes at early stages of development. Large larval populations can be sorted into populations of males/females; transgenics/non-transgenics, heterozygous/homozygous, transgenic females/non-transgenic males. Further, the property of the system to quantify transgene copy number offers a new approach for mosquito sexing based on X-linked insertions at any stage of larval development. The sorting procedure has no impact on the mating ability of the resulting adult males. The system, here tested on *A. gambiae* mosquitoes, could be easily adapted to all mosquito species that are amenable to transgenesis.

## Methods

### Mosquito strains and rearing

*Anopheles gambiae* mosquitoes (G3 and Ngousso strains and their transgenic derivatives) were maintained at 28°C and 70-80% humidity in a 12/12 h day/night cycle. Anesthetized CD1 mice were used for blood feeding and larvae were fed finely ground Tetra Goldfish food (Tetra, Germany). The *DSX* transgenic strain, obtained in the G3 background, has been previously described [18].

The *FK* transgenesis plasmid was assembled by recombining three entry plasmids and one piggyBac destination plasmid using three-fragment Multisite Gateway cloning (Invitrogen) according to the manufacturer’s instructions. The resulting construct contained the *attB1*, *attB2*, *attB3* and *attB4* Gateway seam sites that delimit each of the three cassettes contributed by the entry plasmids (for the full annotated construct sequence refer to Additional file 1). The *Vitellogenin* (*AGAP004203*) promoter was amplified from *A. gambiae* genomic DNA using the following primers: 5’-TGACCTCGAGTTCAACTCGACC-3’ and 5’-GATATCGATGGTTCGGTTGTTCGCAGTTG-3’. The amplified fragment was cloned into Xho I and Cla I restriction sites of the *YFP*-containing entry vector. The *AGAP002620* promoter region was amplified using primers: 5-CCGTCTAGACCGGGCTCTACAAAGTC-3’ and 5’-CAGCTCTCGAGCAGGAGGATCGTT-3’ and cloned as an Xba I - Xho I fragment into the *tdTomato*-containing entry vector. Embryos (n = 120) of the Ngousso strain were injected with a 200 ng/μl solution of the transgenesis plasmid and 20 surviving adults were back-crossed to Ngousso mosquitoes. A single transgenic mosquito male was recovered from the back-cross progeny. Further genetic crosses revealed that the transgene insertion was X-linked. The piggyBac insertion was mapped by inverse PCR as follows: 500 ng of genomic DNA were digested with *Sau*3AI or cocktails of blunt end restriction enzymes (*Sca*I *Hinc*II, *Dra*I, *Sma*I *Pvu*II, Fermentas), and re-ligated with T4 DNA ligase (Fermentas) in a final volume of 500 μl. The sample was ethanol-precipitated, re-suspended in 20 μl water, of which 2 μl were subjected to PCR. The piggyBac 5’ border of the insertion site was mapped by sequencing a product amplified with primers 5’-TGCACAGCGACGGATTCGCGCTATT-3’and 5’-AGGACATCTCAGTCGCCGCTTGGA-3’, followed by nested PCR with 5’-CGCGCTATTTAGAAAGAGAGAG-3’ and 5’-GAACTATAACGACCGCGTGAGTC-3’; or with 5’-GAACTATAACGACCGCGTGAGTC-3’ and 5’-CAGTGACACTTACCGCATTGACA-3’. The piggyBac 3’ border of the insertion site was mapped by sequencing the product amplified with primers 5’-CGAGGTTTATTTATTAATTTGAATAGATATTAAG-3’ and 5’-CGATATACAGACCGATAAAACACATGCGT-3’, followed by nested PCR with 5’-GCGTCAATTTTACGCATGATTATCTTT-3’ and 5’-ATTTACACTTACATACTAATAATAAATTCAAC-3’.

Amplified fragments were compared by BLAST to the *Anopheles gambiae* genome (VectorBase). The transposon insertion was mapped within a 232-base pair repeated element on the X chromosome. This short repeated element is broadly distributed in the genome, but at the position X: 22463464 is present in the Ngousso strain and absent from the G3 and PEST strains. The transgenic construct carried the *EGFP* gene under the control of a *3xP3* promoter [19] as a transgenesis selection marker, and two additional reporter genes: (i) *YFPvenus*[20] under the control of the *A. gambiae Vitellogenin* (*AGAP004203*) promoter and (ii) *tdTomato*[21] under the control of the *AGAP002620* gene promoter [22]. The detailed characterization of these additional reporter constructs will be described elsewhere. Mosquitoes were reared and blood-fed on anesthetized mice in compliance with French and European laws on animal house procedures (agreement #E67-482-2 of the Direction of Veterinary services of the French Ministry of Agriculture).

### COPAS-assisted larval sorting

The blood-fed mosquitoes (two to four days after a blood meal) were offered an egg dish made of a conical, 90 mm diameter, hardened filter paper (#50 Whatman, GE Healthcare, UK) dipped in a glass bowl filled with water. Larvae were allowed to hatch directly in the egg dish and were recovered from water leaving most of the empty eggshells on the filter paper. Larvae were transferred to the reservoir of a Complex Object Parametric Analyzer and Sorter (COPAS) large particles flow cytometry *SELECT* instrument (Union Biometrica, Holliston, MA, USA), and analysed with the Biosort5281 software. For laser-assisted sorting, 488 or 514 nm emission filters were used indifferently with the following acquisition parameters: Green PMT 500, Yellow PMT 500, Red PMT 600, Delay 8; Width 6, pure mode selection with superdrops. Detection thresholds were set to 100 (signal) and 150 (time of flight). “Sheath” pressure (demineralized water was used instead of Sheath medium) was kept at a value of around 3, sorter pressure 3.3, while sample pressure varied between 3 and 4.5 depending on the density of the larvae. The flow rate was kept below 18 detected objects per second. Selected larvae were collected into a Petri dish placed underneath the flow cell outlet. Though result diagrams can be directly viewed in the COPAS® software, the LMD files generated by the COPAS analysis were imported in the WinMDI freeware for data analyses and diagram generation.

### Fluorescence microscopy

Small larvae were spotted in a drop of water in the wells of a CEL-LINE teflon-coated, 24-well diagnostic microscope slide (Erie Scientific, Menzel GmbH, Braunschweig; Germany) and observed with the 5x objective of an Axiovert 200 M Zeiss fluorescence microscope. To immobilize larvae for photography, an anaesthetics solution of final concentration 5% tricaine and 0.5% tetramizole was added to the water 15 min before observation.

## Results

### Rapid and precise establishment of homozygous transgenic *Anopheles gambiae* lines by COPAS sorting

**Figure 1:**
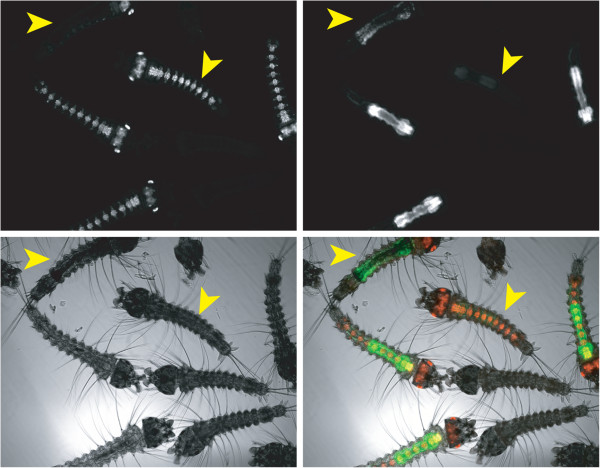
**Sex-specific expression of the*Dsx-GFP*transgene.** A mix of wild-type and heterozygous *DSX* first instar larvae observed under a fluorescence microscope with a 5x objective. Red fluorescence (upper panel, left) denotes larvae carrying the *DsRed* transgene (the upper larva shows its dorsal side, the four additional red larvae show their ventral side). Green fluorescence intensity (upper panel, right) allows distinguishing between females (less bright, arrowheads) and males (showing stronger fluorescence). Lower panels: left, transmission image; right; overlay of images on upper panels.

**Figure 2:**
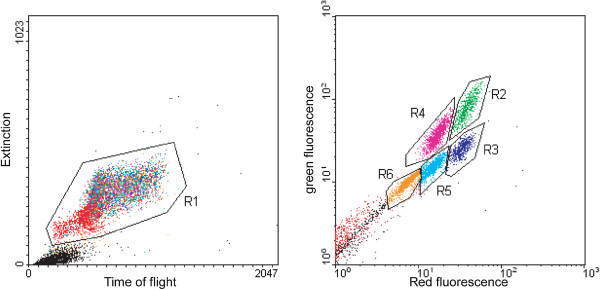
**COPAS-assisted analysis of larval populations.** The progeny of *DSX*/+ mosquitoes, containing non-transgenic as well as heterozygous and homozygous transgenic mosquitoes, was analysed by COPAS. The left diagram (Extinction *vs* Time of Flight) shows all detected objects. Mosquito larvae were empirically determined to be located inside the gated area (R1). The right diagram (red *vs* green fluorescence) decomposes the larval population into five categories: non-transgenic (+/+, R6), heterozygous females (*XX; DSX/+*, R5), heterozygous males (*XY; DSX/+*, R4*),* homozygous females (XX; *DSX*, R3), homozygous males (XY; *DSX*, R2), as indicated.

The goal of this work was to evaluate the possibility of performing accurate, fast and high-throughput larval screening and sorting using a flow cytometry machine, the Complex Object Parametric Analyzer and Sorter (COPAS®, Union Biometrica). To this end, the *DSX::EGFP* transgenic line (thereafter, *DSX*) [18] was used, as it comprises a combination of fluorescent markers that allow the differentiation between male and female larvae as early as the first instar stage (based on higher expression levels of an *EGFP* reporter gene in the male midgut), and between heterozygous and homozygous individuals (based on higher expression levels of the selectable DsRed marker in the central nervous system of homozygotes) (Figure 1). To test the efficacy and sensitivity of the COPAS machine to sort different classes of larvae, non-transgenic mosquitoes were crossed to *DSX* mosquitoes and the resulting F_2_ larvae were analysed. Such progeny was expected to segregate into five fluorescence classes according to single gene Mendelian inheritance and sex-specific expression of the GFP marker: 12.5% of homozygous transgenic females, 12.5% of homozygous transgenic males, 25% of heterozygous females, 25% of heterozygous males, and 25% of homozygous non-transgenic males and females (for which no fluorescence-based sex separation was possible). First instar larvae were COPAS-analysed and the number and ratio of larval classes were quantified by COPAS software. First, the area corresponding to live larvae was determined using light extinction and time of flight parameters. Signals beyond this area were identified by microscopy as eggshells and debris from the larval breeding water. The selected area was next analysed by fluorescence and the five expected larval classes were identified (Figure 2). Out of 4,036 larvae screened, 493 (12.2%) showed high green (EGFP-positive) and high red (DsRed-positive) fluorescence (homozygous transgenic males), 520 (12.9%) were low green and high red (homozygous transgenic females), 1,003 (24.9%) were high green and low red (heterozygous transgenic males), 958 (23.7%) were low green and low red (heterozygous transgenic females) and 1,062 (26.3%) were negative for any fluorescence (Figure 2). These values are consistent with the expected percentages (goodness of fit χ^2^ =6.072, p = 0.19). About 500 larvae from each of the four transgenic populations were sorted separately (in less than 30 min) and the accuracy of sex sorting was verified by visual examination of the resulting adults. All individuals were of the predicted sex. 

Next, male and female homozygous transgenic adults obtained from the sorting process were crossed and their progeny was screened (again using the COPAS instrument) to search for potential heterozygous individuals arising from inefficient sorting of homozygotes. Heterozygotes were absent, suggesting that the initial sorting of homozygous individuals by COPAS was 100% efficient, thereby permitting the establishment of a homozygous transgenic mosquito line in one generation. These results demonstrate the potential of the COPAS machine for compensating the lack of balancer chromosomes in mosquitoes for selection and maintenance of a desired transgenic genotype, thereby permitting a major gain in time and precision.

### COPAS-assisted high-throughput sexing of early mosquito larvae

**Figure 3:**
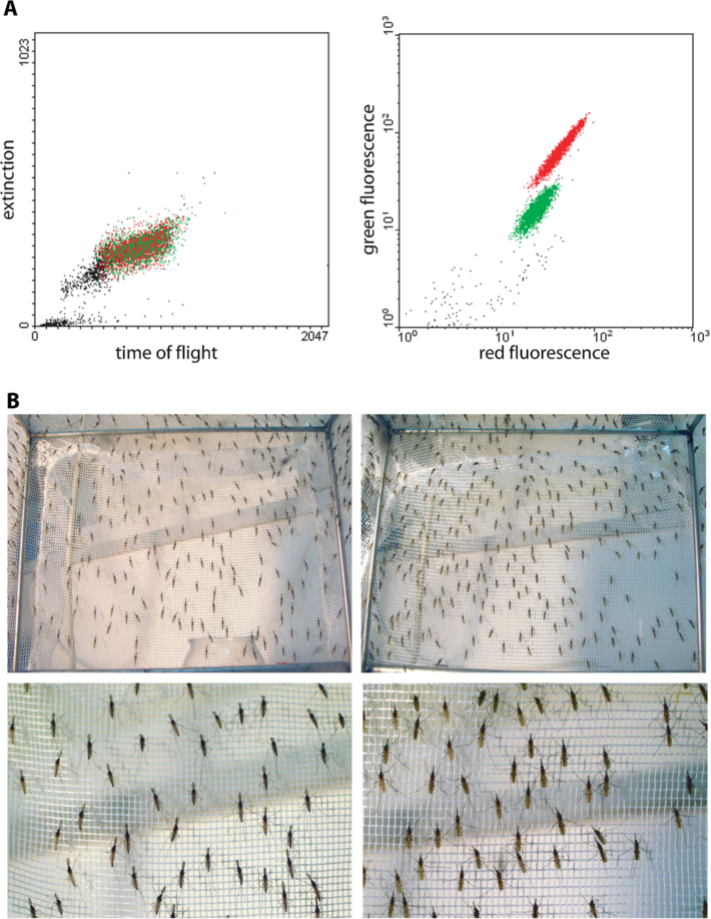
**COPAS profile of the homozygous*DSX*strain and sexual selection.A.** About 5,000 freshly hatched larvae of the homozygous *DSX* line were subjected to COPAS analysis and sorting. The data generated by COPAS was treated with WinMDI software to analyse the results and to express results as artificially colored regions corresponding to males (red) and to females (green) on the fluorescence diagram (on the right). Dot colours are the same as in the time-of-flight *vs* extinction diagram (left). **B.** A total of 2,000 larvae of each sex were selected with COPAS and grown to adulthood. Careful visual examination of the cages did not reveal the presence of any adult of the wrong sex. Left panels: male mosquitoes, as identified by the hairy antennae; right panels: female mosquitoes. Bottom panels are close-ups of top panels.

To test the efficiency and reliability of sorting single-sex mosquito populations, first instar homozygous *DSX* larvae were analysed with the COPAS machine, which detected two distinct populations of larvae displaying high and low levels of green fluorescence that were expected to correspond to male and female populations, respectively (Figure 3A).

After sorting, 2,000 larvae of each population were raised separately to the pupal stage and resulting pupae were placed in two different cages. Careful and repeated visual examination of the adults emerged in each cage confirmed that all adults from the larvae displaying high GFP fluorescence were males and all adults from the larvae showing low GFP fluorescence were females (Figure 3B).

In an independent experiment, groups of 150 larvae were recovered in four successive passages of the same individuals through the COPAS machine, sorting for male and for female larvae successively. These larvae were raised to adulthood and the sex of each mosquito verified. Again, visual examination confirmed the 100% accuracy of sorting at each of the four passages; none of the sorted groups contained an adult of the wrong sex. Importantly, the number of larvae from the fourth sorting that developed to adulthood (>100 individuals) was similar to the number of larvae that underwent 0, 1, 2 or 3 COPAS treatments (Table 1). These results suggest that the survival rate of COPAS-sorted larvae was not affected by the sorting procedure itself repeated up to three times.

**Table 1:** Efficiency of COPAS sorting and survival of sorted larvae

		**Number of COPAS sortings**			
	0	1	2	3	4
Sorted sex	-	males	females	males	females
Sorted larvae	150	154	150	153	153
Survivors	110	118	117	112	129
Survival rate	73.3%	76.6%	78.0%	73.2%	84.3%
Sorting efficiency	100%	100%	100%	100%	100%

A population of newly hatched *DSX* larvae was passed through the COPAS machine the indicated number of times and larvae of the indicated sex were requested successively. The number of larvae surviving to adulthood was scored and the sex of the resulting adults was verified. No error was found and survival was not affected by the number of passes through the machine.

### COPAS sorting does not impair male mating competitiveness

The next step was to examine whether COPAS treatment negatively affected fitness and reproductive capability of sorted male insects. To this end, the reproductive success of males raised in standard conditions was compared to that of males that had passed three times through the COPAS machine. Two crosses were assembled in separate cages. Each cage contained 100 non-fluorescent, wild-type virgin females, 100 non-fluorescent competitor males, and 100 *DSX* transgenic males that were either untreated or had been sorted three times with COPAS at the early larval stage. Freshly emerged mosquitoes of each kind were simultaneously placed in the two cages and kept together for five days. Females received a blood meal on day 5, and on day 8 they were isolated into single plastic tubes to oviposit on shallow water. Freshly hatched larvae from individual females were examined under the fluorescence microscope to score the identity of their father. No significant difference was detected in the number of progeny fathered by the COPAS-treated *vs* control *DSX* males (Table 2), suggesting that COPAS sorting does not impair male competitiveness. Note that these experiments revealed a significant proportion of females fertilized by more than one male (15 progenies, out of 80 analysed, arose from females inseminated by at least two males of different genotypes, suggesting an overall rate of multiple matings in these experiments of at least 37.5%). This result is in agreement with previous laboratory-based reports showing that multiple inseminations occur in crowded cages [23], possibly due to repeated female exposure to males in the first 24 h after mating before the mechanisms of refractoriness to further matings are fully activated. 

**Table 2:** Effect of COPAS on male reproductive competitiveness

	**Non-sorted*DSX*males**	**Sorted*DSX*males**
Progenies of *DSX* male	18	19
Progenies of a competitor male	15	13
Mixed Progenies	8	7
Total number of progenies	41	39

Two crosses were assembled in separate cages. Each cage contained 100 wild-type virgin females, 100 wild-type competitor males, and 100 *DSX* males that were either raised normally (non-sorted *DSX* males) or sorted three times with the COPAS (sorted *DSX* males). Freshly emerged mosquitoes of each kind were simultaneously placed in the cages and kept together through the blood meal on day 5 until day 8, when females were isolated into single plastic tubes to oviposit on shallow water. Freshly hatched larvae were examined under the fluorescence microscope to score the identity of their father (100% fluorescent progeny indicated a *DSX* male, 100% non-fluorescent a competitor male, mixed progenies arose from females fertilized by at least two males of different genotypes). Results from the two crosses are not statistically different (χ^2^ = 1.053, p = 0.5906). Several repeats of this experiment confirmed that COPAS-sorted males are not noticeably inferior in their reproductive competitiveness.

Three additional independent repeats of this experiment were performed with smaller mosquito numbers (45–60 mosquitoes for each group). These confirmed the absence of significant loss of fitness and mating competitiveness for males that passed through the machine compared to control males of the same genotype (data not shown). These results suggest that repeated COPAS sorting does not confer any obvious mating disadvantage to the males, at least in laboratory conditions.

### Isolation of non-transgenic male-only populations from transgenic colonies

**Figure 4:**
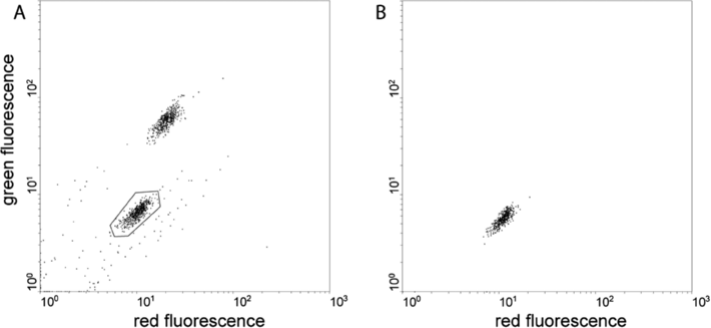
**COPAS-assisted sorting of a pure population of non-transgenic mosquito males.A.** About 1,000 freshly hatched larvae of the progeny of a cross between males of the *FK*^*X*^ line (having a GFP transgene inserted on the X chromosome) and wild type females were subjected to COPAS sorting. The lower area (gated) corresponds to wild-type male larvae, the upper area to heterozygous transgenic females. **B.** The 437 male larvae sorted in A were analysed again by the COPAS to assess the occurrence of female contamination.

For certain types of vector control interventions (release of sterile males for non-transgenic SIT or release of selected natural disease-resistance traits), it will be desirable to obtain large populations of non-transgenic mosquito males. This would be essential in all instances where the release of transgenic insects may not be possible for regulatory reasons. A simple COPAS-based strategy was designed to obtain a large, non-transgenic, male-only population based on the inheritance of an X-linked gene in the F_1_ generation. This strategy made use of a newly established transgenic mosquito line, the *FK*^*X*^ line, carrying a GFP-expressing transgene on the X chromosome (see Methods). A total of 120 *FK*^*X*^ males were crossed to 200 non-transgenic virgin females. In the F_1_ progeny, all males inherit their fathers' Y chromosome and are non-transgenic, while all females inherit a copy of the GFP-expressing X chromosome. Taking advantage of this property, the COPAS sorter was set to select only the non-fluorescent F_1_ male larvae (Figure 4A). For quality control, the sorted individuals were immediately re-analysed by the COPAS. All objects fell in the non-fluorescent region of the diagram, confirming the absence of any contaminating transgenic female larvae (Figure 4B). Visual examination of the resulting cage of adult males confirmed their purity. Therefore, the use of COPAS allows the selection of non-transgenic, male-only larvae from a cross between non-transgenic females and males from an X-linked transgenic line. The limiting step of this procedure for high-throughput applications is the separation of female-only wild type individuals for the crosses: this limitation would be overcome by the generation of Y-linked transgenic lines allowing to sort (again by the use of fluorescence-assisted sorting) large numbers of non-transgenic virgin females from their transgenic siblings.

## Discussion

The possibility to employ genetic control strategies to roll back malaria and other mosquito-borne diseases calls for new technological approaches that overcome the current blocks in mass-rearing of mosquitoes with the desired phenotypes. The work reported here demonstrates that the technology of large particle flow cytometry provides an extremely efficient, fast and reliable method for the selection of male-only (or female-only) mosquito populations early in their developmental cycle, and for the rapid and effective establishment of homozygous transgenic lines. This work builds on an earlier report that provided a proof-of-principle demonstration of the potential of COPAS for genetic sexing of mosquito larvae [17]. This initial study was restricted to the separation of the sexes and achieved incomplete accuracy and low speed of sorting due to the advanced stage of larvae development. Here, these limitations are overcome by exploiting a sex-specific fluorescent marker expressed at the very early stages of larval development. Moreover, it is shown here that X-linked transgenes efficiently substitute for early sex-specific markers for mosquito sexing. Currently, sexing in *Culicidae* is achieved manually by microscopic sorting of pupae based on the dimorphism of the male and female terminal abdominal segments. Another method for separation of the sexes exploits differences in size of male and female pupae characteristic of *Aedes* mosquitoes (http://www.oxitec.com/news-and-views/topic-pages-safety-and-sustainability/what-happens-if-female-mosquitoes-are-released/). This approach cannot be used for *A. gambiae*, a mosquito species in which the size difference between male and female pupae is too subtle to warrant accurate separation of females from males. Therefore, to date COPAS sorting is the only reliable method that allows high-throughput sexing in *Anopheles*.

The performance of the current COPAS sorters (40,000 male larvae per hour) would allow the separation of almost 2 million male mosquitoes per week (considering a 20% larval mortality rate and an 8-hour daily use of a single machine). Based on past SIT attempts [24], this number would be sufficient for most release strategies. For interventions requiring larger number of released males, the sorting method described here would need to be scaled up. The further improvement of sorters specifically designed for mosquitoes (upgraded COPAS instruments or similar sorters that may be developed by other manufacturers) may render this task achievable in the near future.

Ordinarily when a new transgenic mosquito line is obtained, inaccurate and lengthy visual screening of larvae from a heterogeneous F_2_ population must be performed by fluorescence microscopy to select homozygous individuals based on their stronger fluorescence phenotype. This process is subjective and prone to human error; in addition, given the limited speed of the manual process, only a small number of homozygous larvae can be obtained by one operator in a few hours. These limitations make obtaining stable homozygous transgenic lines a lengthy, tedious and inefficient process. As illustrated here for the *DSX* line, COPAS usage revolutionizes this step and yields a large population of homozygous individuals in one sorting session lasting less than 30 min, based on the differential expression level of the selectable marker in heterozygous and homozygous individuals. This method, now routinely used in the laboratory, facilitated the establishment of more than 20 distinct transgenic lines in two distinct *A. gambiae* genetic backgrounds, the G3 and Ngousso strains (data not shown), illustrating its general value for the development of transgenic mosquito lines.

The availability of an effective and high-throughput technology to rapidly select homozygous individuals from mixed populations offers the possibility to increase the fitness of released insects. It is particularly important when considering the problems posed by mass-rearing transgenic insects, where inbreeding of the population causes large fitness costs. As a way to increase the genetic pool of inbred populations with a consequent positive effect on fitness, field mosquitoes can be introduced into the mass-reared population every few generations. Moreover, the sorting technology allows purifying a transgenic line in case of contamination with wild-type individuals, a frequently occurring problem even in the most experienced laboratories.

The selection procedure described here and the use of multiple fluorescent markers such as GFP, YFP, CFP and DsRed proteins in transgenic larvae enable novel experimental schemes now routinely used by the authors. For example, multiple transgenes can be rapidly combined into a single mosquito line, or the frequency dynamics of multiple transgenes present in a single population can be monitored over generations and corrected at will.

When using transgenic lines that allow separation of the sexes based on fluorescent phenotypes, COPAS-assisted sexing can produce large single-sex mosquito populations. In addition to enabling particular laboratory studies that require considerable numbers of virgin females or males, such a tool will be invaluable for the selection of male-only mosquito populations for genetic control strategies against vector-borne diseases such as malaria. In the current experiments, a limitation on the number of screened individuals (up to 20,000 in 30 min) is only imposed by the size of the mosquito colony and not by the throughput characteristics of the COPAS instrument used. Importantly, COPAS-selected males are as competitive for mating as their unsorted peers in cage conditions. In addition, the authors’ empirical experience in managing more than 20 independent transgenic *A. gambiae* strains using routine COPAS sorting indicates that sorted populations are often healthier than their unsorted peers, possibly because COPAS sorting eliminates microbe-rich debris from progenitor mosquitoes.

## Conclusions

The powerful capacity of the COPAS system to distinguish gene copy number by intensity of fluorescence is not limited to the use of DSX-like sex-specific constructs, and is applicable to a large number of insect species that are amenable to transgenesis. Novel COPAS-based strategies will certainly further assist the development of transgenic applications, such as large-scale larval seeding in multi-well plates for high-throughput drug or small molecule screening.

We are now entering a new era in which much anticipated genetics-based disease control strategies are finally taking shape. Considerable laboratory work is urgently needed to evaluate their promises and risks, and to determine the practical aspects of their implementation. The tools presented here remove many of the hurdles related to inaccurate and low-throughput sorting of mosquito populations and thereby pave the way for both laboratory studies and future field releases of sterile or disease-resistant vectors.

## Competing interests

The authors declare that they have no competing interests.

## Authors’ contributions

CS and FC designed and constructed the DSX plasmid and the transgenic mosquito strain. EM designed methods, performed experiments and drafted the manuscript. JS and CK generated and mapped the *FK*^*X*^ line, respectively. EAL and FC conceived the study, participated to its design and co-ordination and drafted the manuscript. All authors read and approved the final manuscript.

## Supplementary Material

Additional file 1 **Description of the FK**^**X**^**transgenic construct.** Click here for file
