# Supplementary material for: High-throughput sorting of mosquito larvae for laboratory studies and for future vector control interventions
Source: Malar J. 2012 Aug 28;11:302. doi: 10.1186/1475-2875-11-302 (PMC3470999; doi:10.1186/1475-2875-11-302)
Supplement: Additional file 1 — Description of the FKXtransgenic construct. [file 1475-2875-11-302-S1.docx]

**Supplemental Text**

**Description of the FK^X^ transgenic construct**

The full nucleotide sequence of the piggyBac construct inserted in the Ngousso strain of *A. gambiae* is shown below in annotated Genbank format, along with a stretch of the genomic sequences flanking the insertion site on the X chromosome.

LOCUS FK annotated 10481 bp DNA linear

FEATURES Location/Qualifiers

misc_feature 9924..10481

/note="flanking chromosomal sequence, 3' side"

misc_feature complement(9641..9673)

/note="loxP"

misc_feature 9674..9923

/note="piggyBac vector, 3' sequence"

misc_feature complement(9163..9594)

/note="gypsy insulator from D. melanogaster"

misc_feature complement(9132..9153)

/note="attB1"

misc_feature 8315..9102

/note="drosomycin terminator from D. melanogaster"

misc_feature 6867..8295

/note="dTomato"

misc_feature 5352..6847

/note="AGAP002620 promoter region"

misc_feature 5324..5350

/note="attB4"

gene 4341..5057

/note="EYFP (Clontech)"

misc_feature 5064..5320

/note="SV40 term sequences"

misc_feature 2636..4337

/note="Vitellogenin (AGAP004203) promoter"

misc_feature complement(2612..2634)

/note="attB3"

misc_feature complement(2371..2576)

/note="3xPax3-hsp70 promoter"

misc_feature complement(1632..2351)

/note="GFP"

misc_feature complement(1393..1614)

/note="SV40 term sequences"

misc_feature complement(889..1319)

/note="gypsy insulator from D. melanogaster"

misc_feature 1330..1350

/note="attB2"

misc_feature complement(594..814)

/note="phage PhiC31 attP'"

misc_feature complement(825..858)

/note="loxP"

misc_feature 284..593

/note="piggyBac vector, 5' sequence"

misc_feature 1..283

/note="flanking chromosomal sequence, 5' side"

ORIGIN

1 CCCGGGAATG GGAGTGGTAT GGGTGAAAAA GAATCCAAGA GCGTGTGTTT CTACGCTGGA

61 GTAAATATAT AAAAGCCGTT TCAAAAAGGG TGCCGTAGAC TTCTTATTCT TAGTGTTGGA

121 AACTCGTGAG TGCCCAACAG TCTGTCAGGT GACAACGCTA GAGAAGAGAG TGAGAGGGAG

181 GATAAAAACG GTAAATTAGG AGATAAGGAG AAGGGTTGGT CCGCTCCTAA CCAGTTAGTT

241 GAAATTCGAC AATCGGTACA AGTGGACTAA TTTTTGGATT TAAccctaga aagatagtct

301 gcgtaaaatt gacgcatgca ttcttgaaat attgctctct ctttctaaat agcgcgaatc

361 cgtcgctgtg catttaggac atctcagtcg ccgcttggag ctcccgtgag gcgtgcttgt

421 caatgcggta agtgtcactg attttgaact ataacgaccg cgtgagtcaa aatgacgcat

481 gattatcttt tacgtgactt ttaagattta actcatacga taattatatt gttatttcat

541 gttctactta cgtgataact tattatatat atattttctt gttatagatt agatcgcgct

601 cgcgcgactg acggtcgtaa gcacccgcgt acgtgtccac cccggtcaca accccttgtg

661 tcatgtcggc gaccctacgc ccccaactga gagaactcaa aggttacccc agttggggca

721 ctactcccga aaaccgcttc tgacctggga aaacgtgaag ccccggggca tccgctgagg

781 gttgccgccg gggcttcggt gtgtccgtca gtactgcagg taccataact tcgtatagca

841 tacattatac gaagttatac ctcccaacgc gttggatgca gcactagagt tgttggttgg

901 cacaccacaa atatactgtt gccgagcaca attgatcggc taaatggtat ggcaagaaaa

961 ggtatgcaat ataataatct tttattgggt atgcaacgaa aatttgtttc gtcaacgtat

1021 gcaatattyt ttattaaaag agggtatgca atgtatttta ttaaaaacgg gtatgcaata

1081 taataatctt ttattgggta tgcaacgaaa atttgtttcg tcaaagtatg caatattttt

1141 tattaaaaga gggtatgcaa tgtattttat taaaaacggg tatgcaataa aaaattattt

1201 ggtttctcta aaaagtatgc agcacttatt ttttgataag gtatgcaaca aaattttact

1261 ttgccgaaaa tatgcaatgt ttttgcgaat aaattcaacg cacacttatt acgtggccaa

1321 ctagatcaac cactttgtac aagaaagctg ggtactcgag ctccaccgcg gtggcggccg

1381 ctctagctaa gatacattga tgagtttgga caaaccacaa ctagaatgca gtgaaaaaaa

1441 tgctttattt gtgaaatttg tgatgctatt gctttatttg taaccattat aagctgcaat

1501 aaacaagtta acaacaacaa ttgcattcat tttatgtttc aggttcaggg ggaggtgtgg

1561 gaggtttttt aaagcaagta aaacctctac aaatgtggta tggctgatta tgatctagag

1621 tcgcggccgc tttacttgta cagctcgtcc atgccgagag tgatcccggc ggcggtcacg

1681 aactccagca ggaccatgtg atcgcgcttc tcgttggggt ctttgctcag ggcggactgg

1741 gtgctcaggt agtggttgtc gggcagcagc acggggccgt cgccgatggg ggtgttctgc

1801 tggtagtggt cggcgagctg cacgctgccg tcctcgatgt tgtggcggat cttgaagttc

1861 accttgatgc cgttcttctg cttgtcggcc atgatataga cgttgtggct gttgtagttg

1921 tactccagct tgtgccccag gatgttgccg tcctccttga agtcgatgcc cttcagctcg

1981 atgcggttca ccagggtgtc gccctcgaac ttcacctcgg cgcgggtctt gtagttgccg

2041 tcgtccttga agaagatggt gcgctcctgg acgtagcctt cgggcatggc ggacttgaag

2101 aagtcgtgct gcttcatgtg gtcggggtag cggctgaagc actgcacgcc gtaggtcagg

2161 gtggtcacga gggtgggcca gggcacgggc agcttgccgg tggtgcagat gaacttcagg

2221 gtcagcttgc cgtaggtggc atcgccctcg ccctcgccgg acacgctgaa cttgtggccg

2281 tttacgtcgc cgtccagctc gaccaggatg ggcaccaccc cggtgaacag ctcctcgccc

2341 ttgctcacca tggtggcgac cggtctcgag ccgattgttt agcttgttca gctgcgcttg

2401 tttatttgct tagctttcgc ttagcgacgt gttcactttg cttgtttgaa ttgaattgtc

2461 gctccgtaga cgaagcgcct ctatttatac tccggcggtc gagggttcga aatcgataag

2521 cttggatcct aattgaatta gctctaattg aattagtctc taattgaatt agatccgggc

2581 ccggtactcg acgctagcgg ggggcccggt accaacttta ttatacaaag ttgtctcgag

2641 ttcaactcga ccaataataa ttgatccgtc aatccatatt ggtccgcaat aatgaaagtt

2701 gcaagagtac gacggtatga aaagaggttc agtaagttgt aaactaatag tttcttccca

2761 acgttcaaat gctggcaaat cttttcgcgg gccgcacttc gtcgatcgct agtcttaatg

2821 ataatttctg agaaaaaggt gctactgcat ctactatatt ctactggata taaatgaaat

2881 aacaacgtga gactcaccta caacatgtaa tttattgatg gtttagttta accaacctat

2941 gaaataattt gatatagaaa tttgtagtcg tttttctatg aagtaaaatt ctaaaatcaa

3001 acattaaact gttttgtagt acccggactc atggtatggc ttctattagc cgtaaacaaa

3061 gatttacaat tgactaaggt taggtccgac actgtaggag ccagcgcgtc ctttcaatac

3121 atcaacggac catctcgtgt tgttaaatac ttattattat tatggtttgc taattgatat

3181 gttccaagac cgatttggat ttcgaaataa gtattctctg attcattttg ggagccggtc

3241 tcgtgataca gtcgtcagcc cgtacgactt aactacattc tcgtcatggg ttcaagcccc

3301 agatggaccg tgccgccata cgtagagtca gtcctatcct gttatggggg gtaatacata

3361 agacactgaa agccaacccc acaagtggta cagacaagcc ttgccgacaa ttgttgttga

3421 gccaaacaga agaagaatcc atttcgggaa atgattttat cattcaatca aaccagtcaa

3481 tcataaacat catagtttta aatactcaaa actagttgag atctttaaaa cacattattt

3541 tagtttaatt aaatgatctg ttagctagaa ggtagatacg atattttaga catttcgtaa

3601 tagatcgcaa atctctatta tgttggtaat tcacttcgta aaactcttag gcaaaactct

3661 tattagtaaa caaaatacta atcaaacact gataaactaa cgcgatttat acattggaca

3721 aagaagaggc tgattttaaa aatactcgct ttaaaatttg cttcattcat caatgtattg

3781 taaagcacat aaagaacaca atcattgact gaaaacaatt ccacgtctca gccaacttcc

3841 aggatcaatg aaatagcaag ttccaagttc catttcattg attatggtaa ctactgatta

3901 ttttcaataa caaatacttc gaagactgca caattcaaaa gtatgccaga aagaaaggat

3961 tactatcaat tgtgggttaa tcaaactaag acaggtggca aaaatggaac cattgattaa

4021 ggcagccact gaccgatttc atttaaaaaa cacactcttg gaagtttcca cacaatctca

4081 ccttttgcca attttagcaa agacgttgtg ctgcactgat aagaatcgaa ctgtaaacat

4141 gtgggcagta aaaattattt catcgttcaa cacggcggtc attacactat tcgaagcagc

4201 tgaaaagatt tgatgatagc aggaccgtga gatcagcaaa tttgaggtat aaaagatgat

4261 cctgcgacca ccagaaggca cattcgagct ttggagtgca ttcaaagcat ccgggcaact

4321 gcgaacaacc gaaccatcgc atggtgagca agggcgagga gctgttcacc ggggtggtgc

4381 ccatcctggt cgagctggac ggcgacgtaa acggccacaa gttcagcgtg tccggcgagg

4441 gcgagggcga tgccacctac ggcaagctga ccctgaagtt catctgcacc accggcaagc

4501 tgcccgtgcc ctggcccacc ctcgtgacca ccttcggcta cggcctgcag tgcttcgccc

4561 gctaccccga ccacatgaag cagcacgact tcttcaagtc cgccatgccc gaaggctacg

4621 tccaggagcg caccatcttc ttcaaggacg acggcaacta caagacccgc gccgaggtga

4681 agttcgaggg cgacaccctg gtgaaccgca tcgagctgaa gggcatcgac ttcaaggagg

4741 acggcaacat cctggggcac aagctggagt acaactacaa cagccacaac gtctatatca

4801 tggccgacaa gcagaagaac ggcatcaagg tgaacttcaa gatccgccac aacatcgagg

4861 acggcagcgt gcagctcgcc gaccactacc agcagaacac ccccatcggc gacggccccg

4921 tgctgctgcc cgacaaccac tacctgagct accagtccgc cctgagcaaa gaccccaacg

4981 agaagcgcga tcacatggtc ctgctggagt tcgtgaccgc cgccgggatc actctcggca

5041 tggacgagct gtacaagtaa agcggccgcg actctagatc ataatcagcc ataccacatt

5101 tgtagaggtt ttacttgctt taaaaaacct cccacacctc cccctgaacc tgaaacataa

5161 aatgaatgca attgttgttg ttaacttgtt tattgcagct tataatggtt acaaataaag

5221 caatagcatc acaaatttca caaataaagc atttttcttc actgcattct agttgtggtt

5281 tgtccaaact catcaatgta tcttaaagct tatcgatacg cgtaccaact ttgtatagaa

5341 aagttgggtg ctcgagcagg aggatcgtta aagcttgttc ctattgcagg catcgtctgt

5401 gttttgtttt aagtatgaca gcttaacgat gcggacgctc tttgttgact gaccgctgct

5461 ttctgcgtcg agtgacattt tgcgggctct ctttcgcata ccgcaacacc acgtacgtag

5521 ccgtctgggt aacgaaaata gcctcaatta gctattgtta gcaagcaatg actaaccgca

5581 attactatca taaaagaaac ggtcgcgcgt caaacaacaa caaacccttt cgatgcgtaa

5641 gagcataatt acacacacac acacacacac actccatggt gcaaatcaat tgagcatcga

5701 tcacaccggc accaggatca cgacaccttt gacccgaagc cggaggtcat actggctgga

5761 gccgagagcc gcaaaaaagc cctttcccca gccttgaggc gccttggtgg cagtatgttg

5821 cgggcaacga tgtaccaacc atgctcctgc aatgtccacc agcatccgca tgtgtgtgtg

5881 tgttgctaaa tggcccccat gaagacgctt gacactacag gctgtatggg tttgttggtt

5941 gtggaattag agggtttcat cggttgcaag ccactactca atccacagag cacgatgaca

6001 tgccgcgaca tgtgtgccaa tttggtgtgc attatccgat actgtacagc ctgccgctgt

6061 cgaaaaatta agcgggcctc ccccctaaat catcggcgac gcgcgatcgt cctcggatgc

6121 catttgctaa ttcgatagtt tcggctaccg acgggtaagc gatggtttga aaaagacgaa

6181 aagaagaagg tatgtagcta ttgtgtagct agaaagagac atacttgcgg ttgcttccct

6241 gcggttggta cgtggtacaa ggtacacctg ccgttggatg cagtggccct cacggagcgg

6301 agcgatcgtt ggggagcttc ttcacacgca cgcacataca aaccgatgtc taatggccag

6361 gttggaaggt acggcaaccg ggtgcgatca acatggaaaa acaatcacaa ctctgcgctg

6421 aaattaattc gtgtgtgtgt gccgcttgct ggctgccatg tatggaaatg cgcgcgtccg

6481 cttgctgtgt gcgcatcttc cgatcgttac acggccgcgt tgagacgaaa ttcgacgcaa

6541 agccgtcgtt cttagtagga caattaactg ccccctctac cctcgccgtc tggctttaac

6601 ggtctctcgc ccccccccgg tacgtgatgt gttgtgcgaa acgatcggtg cccgtatctg

6661 gccggcccga tcggaagcag gggggggggg cattagaggc attagagccg ctcaaggatg

6721 cgtcggaact ggcgcgatgg ttttgttggt ataaatcggt aggaacaatc gtccggtcag

6781 cacattctgt cgtgcggcgt tcgctagtga aggtagaact ttgaagtccg actttgtaga

6841 gcccggtcta gagtcgatcg acaaccatgg tgagcaaggg cgaggaggtc atcaaagagt

6901 tcatgcgctt caaggtgcgc atggagggct ccatgaacgg ccacgagttc gagatcgagg

6961 gcgagggcga gggccgcccc tacgagggca cccagaccgc caagctgaag gtgaccaagg

7021 gcggccccct gcccttcgcc tgggacatcc tgtcccccca gttcatgtac ggctccaagg

7081 cgtacgtgaa gcaccccgcc gacatccccg attacaagaa gctgtccttc cccgagggct

7141 tcaagtggga gcgcgtgatg aacttcgagg acggcggtct ggtgaccgtg acccaggact

7201 cctccctgca ggacggcacg ctgatctaca aggtgaagat gcgcggcacc aacttccccc

7261 ccgacggccc cgtaatgcag aagaagacca tgggctggga ggcctccacc gagcgcctgt

7321 acccccgcga cggcgtgctg aagggcgaga tccaccaggc cctgaagctg aaggacggcg

7381 gccactacct ggtggagttc aagaccatct acatggccaa gaagcccgtg caactgcccg

7441 gctactacta cgtggacacc aagctggaca tcacctccca caacgaggac tacaccatcg

7501 tggaacagta cgagcgctcc gagggccgcc accacctgtt cctggggcat ggcaccggca

7561 gcaccggcag cggcagctcc ggcaccgcct cctccgagga caacaacatg gccgtcatca

7621 aagagttcat gcgcttcaag gtgcgcatgg agggctccat gaacggccac gagttcgaga

7681 tcgagggcga gggcgagggc cgcccctacg agggcaccca gaccgccaag ctgaaggtga

7741 ccaagggcgg ccccctgccc ttcgcctggg acatcctgtc cccccagttc atgtacggct

7801 ccaaggcgta cgtgaagcac cccgccgaca tccccgatta caagaagctg tccttccccg

7861 agggcttcaa gtgggagcgc gtgatgaact tcgaggacgg cggtctggtg accgtgaccc

7921 aggactcctc cctgcaggac ggcacgctga tctacaaggt gaagatgcgc ggcaccaact

7981 tcccccccga cggccccgta atgcagaaga agaccatggg ctgggaggcc tccaccgagc

8041 gcctgtaccc ccgcgacggc gtgctgaagg gcgagatcca ccaggccctg aagctgaagg

8101 acggcggcca ctacctggtg gagttcaaga ccatctacat ggccaagaag cccgtgcaac

8161 tgcccggcta ctactacgtg gacaccaagc tggacatcac ctcccacaac gaggactaca

8221 ccatcgtgga acagtacgag cgctccgagg gccgccacca cctgttcctg tacggcatgg

8281 acgagctgta caagtaagaa ttcgaagctg atccatgagc aattagcatg aacgttctga

8341 aaagcgcgtt tagctctcca ctacttacac atattctatg ctgcaatatt gaaaatctaa

8401 taaacaaaac taatgtacat taattcttca gttttgaata tccttctcct gactttctta

8461 tttagaatta atataatact gcatacatta atactgtaaa tatgataagt acctgcaaaa

8521 cactgcagct caagtcttaa tgaggttctg cgatagctta gcataattag taacttatcg

8581 cgcaggaatt ccctaatgtt cccgacctac atgtacttct gatagttgcc gaggtcaaat

8641 gttgttgtat ttgtattata cctcaatatt ggtatattca atatctaata gtacccaatt

8701 caattgcaaa gatagtcatt aaaaaaacct aaatcacttg caaattgact tttctgccgg

8761 aaaagcaacc ttgacacaca aagttaatta gtttatctgg aagtcatgtg agaaatttgt

8821 aaataaaatt tttcgcagta atttaagtgg gcctaatccc ttttaagcat cttggtttta

8881 cgatgacacc gcaataaggt acaactttat attgtttttg caatcagctt gagtctttat

8941 taggcatcag tctttctctc taagtttctt cgtgcaataa atgaggttcc aaactccgta

9001 gatttttcct tctttgttga atccagatcc tgcaaagaaa aaagagcaaa cccctaggtc

9061 tgtccaggaa tgtattttcg tgtttgtcga tcgaccatgg tctcgacgct agcggggggc

9121 ccggtaccag cctgcttttt tgtacaaact tgttgatcta gagttgttgg ttggcacacc

9181 acaaatatac tgttgccgag cacaattgat cggctaaatg gtatggcaag aaaaggtatg

9241 caatataata atcttttatt gggtatgcaa cgaaaatttg tttcgtcaac gtatgcaata

9301 ttytttatta aaagagggta tgcaatgtat tttattaaaa acgggtatgc aatataataa

9361 tcttttattg ggtatgcaac gaaaatttgt ttcgtcaaag tatgcaatat tttttattaa

9421 aagagggtat gcaatgtatt ttattaaaaa cgggtatgca ataaaaaatt atttggtttc

9481 tctaaaaagt atgcagcact tattttttga taaggtatgc aacaaaattt tactttgccg

9541 aaaatatgca atgtttttgc gaataaattc aacgcacact tattacgtgg ccaactagag

9601 tcgacctcga acgttaacgt taacgtaacg ttaactcgac taacttcgta tagcatacat

9661 tatacgaagt tatgagctca attcgataaa agttttgtta ctttatagaa gaaattttga

9721 gtttttgttt tttttaataa ataaataaac ataaataaat tgtttgttga atttattatt

9781 agtatgtaag tgtaaatata ataaaactta atatctattc aaattaataa ataaacctcg

9841 atatacagac cgataaaaca catgcgtcaa ttttacgcat gattatcttt aacgtacgtc

9901 acaatatgat tatctttcta gggTTAACCT CATACCACAG TAAATAAAGT GTTTTCTCTA

9961 AAAAATCCAC CGCGTTTTTC AACACTTAGA CTTCACTTCT TATCACATAA AGTTCTCACT

10021 ACTCGTAAAA CACAATCAAG AAAGTGTCCC AAAATTAAGA AAACTCCCGA AATCTCTTGT

10081 ATCTATTCGT GAGGTGCATC ATCAGCGTCG TGCGAGTAGC AGCATAATTC ATGGTGCACT

10141 CAGGAAAAAT CATTCCGTGT AACAAACAAC AAGATACAAA AGTACAGGAA GTCCCTGAGA

10201 TACACGGTAC CTCTAATACG CGGAATAGGA GATTCTAAAT ATGACAGTTC TTTGAGCAAA

10261 TTGTACTGAT TTGACTCATC AATTTTTGTC AAATGCAAAA TAATTACCTT TTTGATCGAA

10321 TGTTTAAAAC CAAATCAACA GGATTAAAAC TGTTAAATTT AATCAGACTC ATATCAAATA

10381 ATAGATTACG TGGCTAAAAC CACCCCCTTG CACAATTAAA CGAAATTAGT GATATTTTGG

10441 CTAAAAATTA AGAGGCTCGA CCTACGCAGA AATTCGAGAT A//

**Sequence of the transgenesis vector used to generate the FK^X^ line**

The full annotated sequence of the empty transgenesis vector used to construct the FK reporter transgene is shown below. This vector is a derivative from pXLBacII ([22]: Li et al., Insect Mol Biol 14:1 (17-30), 2005). The *piggyBac transposase* gene under the control of the Drosophila *hsp70* promoter was inserted into the vector backbone (outside of the piggyBac transposon) to serve as a built-in transgenesis helper. A Gateway cassette, genetic insulators and various recombination sites were cloned into the piggyBac region (annotated in the sequence).

LOCUS piggyBac vector, E. Marois 9628 bp DNA circular 01-AUG-2012

FEATURES Location/Qualifiers

misc_feature 6853..6988

/note="PiggyBac 5'region"

misc_feature 3718..3751

/note="loxP"

misc_feature 6458..6678

/note="attP' docking site"

misc_feature complement(339..2405)

/note="piggyBac transposase"

misc_feature complement(2462..3049)

/note="D. melanogaster hsp70 promoter"

misc_feature complement(2407..2462)

/note="rrnB terminator from E. coli"

misc_feature 6414..6447

/note="loxP"

misc_feature 3468..3705

/note="piggyBac 3'region"

misc_feature 3799..4229

/note="gypsy insulator from Drosophila"

misc_feature 4235..5947

/note="Gateway cassette RfB (Invitrogen)"

misc_feature 4239..4363

/note="attR1"

misc_feature 5953..6383

/note="gypsy insulator from Drosophila"

misc_feature complement(5819..5942)

/note="attR2"

ORIGIN

1 CTAAATTGTA AGCGTTAATA TTTTGTTAAA ATTCGCGTTA AATTTTTGTT AAATCAGCTC

61 ATTTTTTAAC CAATAGGCCG AAATCGGCAA AATCCCTTAT AAATCAAAAG AATAGACCGA

121 GATAGGGTTG AGTGTTGTTC CAGTTTGGAA CAAGAGTCCA CTATTAAAGA ACGTGGACTC

181 CAACGTCAAA GGGCGAAAAA CCGTCTATCA GGGCGATGGC CCACTACGTG AACCATCACC

241 CTAATCAAGT TTTTTGGGGT CGAGGTGCCG TAAAGCACTA AATCGGAACC CTAAAGGGAG

301 CCCCCGATTT AGAGCTTGAC GGGGAAAGCC TCGACGGATC CAAATTCAAC AAACAATTTA

361 TGTTTATTTA TTTATTAAAA AAAAACAAAA ACTCAAAATT TCTTCTATAA AGTAACAAAA

421 CTTTTAAACA TTCTCTCCTT TACAAAAATA AACTTATTTT GTACTTTAAA AACAGTCATG

481 TTGTATTATA AAATAAGTAA TTAGTTTAAC TTATACATAA TAGAAACAAA TTATACTTAT

541 TAGTCAGTCA GAAACAACTT TGGCACATAT CAATATTATG CTCTCGACAA ATAACTTTTT

601 TGCATTTTTT GCACGATGCA TTTGCCTTTC GCCTTATTTT AGAGGGGCAG TAAGTACAGT

661 AAGTACGTTT TTTCATTACT GGCTCTTCAG TACTGTCATC TGATGTACCA GGCACTTCAT

721 TTGGCAAAAT ATTAGAGATA TTATCGCGCA AATATCTCTT CAAAGTAGGA GCTTCTAAAC

781 GCTTACGCAT AAACGATGAC GTCAGGCTCA TGTAAAGGTT TCTCATAAAT TTTTTGCGAC

841 TTTGAACCTT TTCTCCCTTG CTACTGACAT TATGGCTGTA TATAATAAAA GAATTTATGC

901 AGGCAATGTT TATCATTCCG TACAATAATG CCATAGGCCA CCTATTCGTC TTCCTACTGC

961 AGGTCATCAC AGAACACATT TGGTCTAGCG TGTCCACTCC GCCTTTAGTT TGATTATAAT

1021 ACATAACCAT TTGCGGTTTA CCGGTACTTT CGTTGATAGA AGCATCCTCA TCACAAGATG

1081 ATAATAAGTA TACCATCTTA GCTGGCTTCG GTTTATATGA GACGAGAGTA AGGGGTCCGT

1141 CAAAACAAAA CATCGATGTT CCCACTGGCC TGGAGCGACT GTTTTTCAGT ACTTCCGGTA

1201 TCTCGCGTTT GTTTGATCGC ACGGTTCCCA CAATGGTTAA CTTATACGGT TCTTGTAGTA

1261 AGTTTTTTGC CAAAGGGATT GAGGTGAACC AATTGTCACA CGTAATATTA CGACAACTAC

1321 CGTGCACAGG CTTTGATAAC TCCTTCACGT AGTATTCACC GAGTGGTACT CCGTTGGTCT

1381 GTGTTCCTCT TCCCAAATAA GGCATTCCAT TTATCATATA CTTCGTACCA CTGTCACACA

1441 TCATGAGGAT TTTTATTCCA TACTTACTTG GCTTGTTTGG GATATACATC CTAAACGGAC

1501 ACCGTCCTCT AAAACCAAGT AACTGTTCAT CTATGGTCAA ATGAGCCCCT GGAGTGTAAT

1561 TTTGTATGCA CTGATGGATA AAGAGATCCC ATATTTTTCT AACAGGAGTA AATACATCGT

1621 TTTCTCGAAG TGTGGGCCGT ATACTTTTGT CATCCATTCT AAGACATCGT ATCAAAAAAT

1681 CAAAACGATC ACGACTCATT ACAGAGACGT ACACCATTGA CAAAGATCGA TCAAAGAGGT

1741 CATCTGTGGA CATGTGGTTA TCTTTTCTCA CTGCTGTCAT TACCAGAATA CCAAAGAAAG

1801 CATAGATTTC ATCTTCATTC GTGTCACGAA ATGTAGCACC TGTCATAGAT TCCCGACGTT

1861 TCAATGATAT CTCAGCATTT GTCCATTTTA CAATTTCCGA AATTATCTCA TCAGTAAAAA

1921 ATAGTTTGAA GCATAAAAGT GGGTCATATA TATTGCGGCA CATACGCGTC GGACCTCTTT

1981 GAGATCTGAC AATGTTCAGT GCAGAGACTC GGCTACGCCT CGTGGACTTT GAAGTTGACC

2041 AACAATGTTT ATTCTTACCT CTAATAGTCC TCTGTGGCAA GGTCAAGATT CTGTTAGAAG

2101 CCAATGAAGA ACCTGGTTGT TCAATAACAT TTTGTTCGTC TAATATTTCA CTACCGCTTG

2161 ACGTTGGCTG CACTTCATGT ACCTCATCTA TAAACGCTTC TTCTGTATCG CTCTGGACGT

2221 CATCTTCACT TACGTGATCT GATATTTCAC TGTCAGAATC CTCACCAACA AGCTCGTCAT

2281 CGCTTTGCAG AAGAGCAGAG AGGATATGCT CATCGTCTAA AGAACTACCC ATTTTATTAT

2341 ATAGGATCCC CGACACCAGA CCAACTGGTA ATGGTAGCGA CCGGCGCTCA GCTGGAATTA

2401 GGCCTTCTAG acagataaaa cgaaaggccc agtctttcga ctgagccttt cgttttattt

2461 gAAATTCCCA ATTCCCTATT CAGAGTTCTC TTCTTGTATT CAATAATTAC TTCTTGGCAG

2521 ATTTCAGTAG TTGCAGTTGA TTTACTTGGT TGCTGGTTAC TTTTAATTGA TTCACTTTAA

2581 CTTGCACTTT ACTGCAGATT GTTTAGCTTG TTCAGCTGCG CTTGTTTATT TGCTTAGCTT

2641 TCGCTTAGCG ACGTGTTCAC TTTGCTTGTT TGAATTGAAT TGTCGCTCCG TAGACGAAGC

2701 GCCTCTATTT ATACTCCGGC GCTCTTTTCG CGAACATTCG AGGCGCGCTC TCTCGAACCA

2761 ACGAGAGCAG TATGCCGTTT ACTGTGTGAC AGAGTGAGAG AGCATTAGTG CAGAGAGGGA

2821 GAGACCCAAA AAGAAAAGAG AGAATAACGA ATAACGGCCA GAGAAATTTC TCGAGTTTTC

2881 TTTCTGCCAA ACAAATGACC TACCACAATA ACCAGTTTGT TTTGGGATTC TAGGGGGATC

2941 GGGGATCAAT TCTAGTATGT ATGTAAGTTA ATAAAACCCA TTTTTGCGGA AAGTAGATAA

3001 AAAAAACATT TTTTTTTTTT ACTGCACTGG ATATCATTGA ACTTATCTGA TCGGCGAACG

3061 TGGCGAGAAA GGAAGGGAAG AAAGCGAAAG GAGCGGGCGC TAGGGCGCTG GCAAGTGTAG

3121 CGGTCACGCT GCGCGTAACC ACCACACCCG CCGCGCTTAA TGCGCCGCTA CAGGGCGCGT

3181 CCCATTCGCC ATTCAGGCTG CGCAACTGTT GGGAAGGGCG ATCGGTGCGG GCCTCTTCGC

3241 TATTACGCCA GCTGGCGAAA GGGGGATGTG CTGCAAGGCG ATTAAGTTGG GTAACGCCAG

3301 GGTTTTCCCA GTCACGACGT TGTAAAACGA CGGCCAGTGA GCGCGCCTCG TTCATTCACG

3361 TTTTTGAACC CGTGGAGGAC GGGCAGACTC GCGGTGCAAA TGTGTTTTAC AGCGTGATGG

3421 AGCAGATGAA GATGCTCGAC ACGCTGCAGA ACACGCAGCT AGATTAACCC TAGAAAGATA

3481 ATCATATTGT GACGTACGTT AAAGATAATC ATGCGTAAAA TTGACGCATG TGTTTTATCG

3541 GTCTGTATAT CGAGGTTTAT TTATTAATTT GAATAGATAT TAAGTTTTAT TATATTTACA

3601 CTTACATACT AATAATAAAT TCAACAAACA ATTTATTTAT GTTTATTTAT TTATTAAAAA

3661 AAACAAAAAC TCAAAATTTC TTCTATAAAG TAACAAAACT TTTATCGAAT TGAGCTCata

3721 acttcgtata atgtatgcta tacgaagtta tGtcgagtta acgttacgtt aacgttaacg

3781 ttcgaggtcg actctagttg gccacgtaat aagtgtgcgt tgaatttatt cgcaaaaaca

3841 ttgcatattt tcggcaaagt aaaattttgt tgcatacctt atcaaaaaat aagtgctgca

3901 tactttttag agaaaccaaa taatttttta ttgcataccc gtttttaata aaatacattg

3961 cataccctct tttaataaaa aatattgcat actttgacga aacaaatttt cgttgcatac

4021 ccaataaaag attattatat tgcatacccg tttttaataa aatacattgc ataccctctt

4081 ttaataaara atattgcata cgttgacgaa acaaattttc gttgcatacc caataaaaga

4141 ttattatatt gcataccttt tcttgccata ccatttagcc gatcaattgt gctcggcaac

4201 agtatatttg tggtgtgcca accaacaact ctagatcaac aagtttgtac aaaaaagctg

4261 aacgagaaac gtaaaatgat ataaatatca atatattaaa ttagattttg cataaaaaac

4321 agactacata atactgtaaa acacaacata tccagtcact atggcggccg cattaggcac

4381 cccaggcttt acactttatg cttccggctc gtataatgtg tggattttga gttaggatcc

4441 gtcgagattt tcaggagcta aggaagctaa aatggagaaa aaaatcactg gatataccac

4501 cgttgatata tcccaatggc atcgtaaaga acattttgag gcatttcagt cagttgctca

4561 atgtacctat aaccagaccg ttcagctgga tattacggcc tttttaaaga ccgtaaagaa

4621 aaataagcac aagttttatc cggcctttat tcacattctt gcccgcctga tgaatgctca

4681 tccggaattc cgtatggcaa tgaaagacgg tgagctggtg atatgggata gtgttcaccc

4741 ttgttacacc gttttccatg agcaaactga aacgttttca tcgctctgga gtgaatacca

4801 cgacgatttc cggcagtttc tacacatata ttcgcaagat gtggcgtgtt acggtgaaaa

4861 cctggcctat ttccctaaag ggtttattga gaatatgttt ttcgtctcag ccaatccctg

4921 ggtgagtttc accagttttg atttaaacgt ggccaatatg gacaacttct tcgcccccgt

4981 tttcaccatg ggcaaatatt atacgcaagg cgacaaggtg ctgatgccgc tggcgattca

5041 ggttcatcat gccgtttgtg atggcttcca tgtcggcaga atgcttaatg aattacaaca

5101 gtactgcgat gagtggcagg gcggggcgta aagatctgga tccggcttac taaaagccag

5161 ataacagtat gcgtatttgc gcgctgattt ttgcggtata agaatatata ctgatatgta

5221 tacccgaagt atgtcaaaaa gaggtatgct atgaagcagc gtattacagt gacagttgac

5281 agcgacagct atcagttgct caaggcatat atgatgtcaa tatctccggt ctggtaagca

5341 caaccatgca gaatgaagcc cgtcgtctgc gtgccgaacg ctggaaagcg gaaaatcagg

5401 aagggatggc tgaggtcgcc cggtttattg aaatgaacgg ctcttttgct gacgagaaca

5461 ggggctggtg aaatgcagtt taaggtttac acctataaaa gagagagccg ttatcgtctg

5521 tttgtggatg tacagagtga tattattgac acgcccgggc gacggatggt gatccccctg

5581 gccagtgcac gtctgctgtc agataaagtc tcccgtgaac tttacccggt ggtgcatatc

5641 ggggatgaaa gctggcgcat gatgaccacc gatatggcca gtgtgccggt ctccgttatc

5701 ggggaagaag tggctgatct cagccaccgc gaaaatgaca tcaaaaacgc cattaacctg

5761 atgttctggg gaatataaat gtcaggctcc cttatacaca gccagtctgc aggtcgacca

5821 tagtgactgg atatgttgtg ttttacagta ttatgtagtc tgttttttat gcaaaatcta

5881 atttaatata ttgatattta tatcatttta cgtttctcgt tcagctttct tgtacaaagt

5941 ggttgatcta gttggccacg taataagtgt gcgttgaatt tattcgcaaa aacattgcat

6001 attttcggca aagtaaaatt ttgttgcata ccttatcaaa aaataagtgc tgcatacttt

6061 ttagagaaac caaataattt tttattgcat acccgttttt aataaaatac attgcatacc

6121 ctcttttaat aaaaaatatt gcatactttg acgaaacaaa ttttcgttgc atacccaata

6181 aaagattatt atattgcata cccgttttta ataaaataca ttgcataccc tcttttaata

6241 aaraatattg catacgttga cgaaacaaat tttcgttgca tacccaataa aagattatta

6301 tattgcatac cttttcttgc cataccattt agccgatcaa ttgtgctcgg caacagtata

6361 tttgtggtgt gccaaccaac aactctagtg ctgcatccaa cgcgttggga gGTataactt

6421 cgtataatgt atgctatacg aagttatGGT ACCTGCAGTA CTGACGGACA CACCGAAGCC

6481 CCGGCGGCAA CCCTCAGCGG ATGCCCCGGG GCTTCACGTT TTCCCAGGTC AGAAGCGGTT

6541 TTCGGGAGTA GTGCCCCAAC TGGGGTAACC TTTGAGTTCT CTCAGTTGGG GGCGTAGGGT

6601 CGCCGACATG ACACAAGGGG TTGTGACCGG GGTGGACACG TACGCGGGTG CTTACGACCG

6661 TCAGTCGCGC GAGCGCGATC TAATCTATAA CAAGAAAATA TATATATAAT AAGTTATCAC

6721 GTAAGTAGAA CATGAAATAA CAATATAATT ATCGTATGAG TTAAATCTTA AAAGTCACGT

6781 AAAAGATAAT CATGCGTCAT TTTGACTCAC GCGGTCGTTA TAGTTCAAAA TCAGTGACAC

6841 TTACCGCATT GACAAGCACG CCTCACGGGA GCTCCAAGCG GCGACTGAGA TGTCCTAAAT

6901 GCACAGCGAC GGATTCGCGC TATTTAGAAA GAGAGAGCAA TATTTCAAGA ATGCATGCGT

6961 CAATTTTACG CAGACTATCT TTCTAGGGTT AATCTAGCTG CATCAGGATC ATATCGTCGG

7021 GTCTTTTTTC CGGCTCAGTC ATCGCCCAAG CTGGCGCTAT CTGGGCATCG GGGAGGAAGA

7081 AGCCCGTGCC TTTTCCCGCG AGGTTGAAGC GGCATGGAAA GAGTTTGCCG AGGATGACTG

7141 CTGCTGCATT GACGTTGAGC GAAAACGCAC GTTTACCATG ATGATTCGGG AAGGTGTGGC

7201 CATGCACGCC TTTAACGGTG AACTGTTCGT TCAGGCCACC TGGGATACCA GTTCGTCGCG

7261 GCTTTTCCGG ACACAGTTCC GGATGGTCAG CCCGAAGCGC ATCAGCAACC CGAACAATAC

7321 CGGCGACAGC CGGAACTGCC GTGCCGGTGT GCAGATTAAT GACAGCGGTG CGGCGCTGGG

7381 ATATTACGTC AGCGAGGACG GGTATCCTGG CTGGATGCCG CAGAAATGGA CATGGATACC

7441 CCGTGAGTTA CCCGGCGGGC GCGCTTGGCG TAATCATGGT CATAGCTGTT TCCTGTGTGA

7501 AATTGTTATC CGCTCACAAT TCCACACAAC ATACGAGCCG GAAGCATAAA GTGTAAAGCC

7561 TGGGGTGCCT AATGAGTGAG CTAACTCACA TTAATTGCGT TGCGCTCACT GCCCGCTTTC

7621 CAGTCGGGAA ACCTGTCGTG CCAGCTGCAT TAATGAATCG GCCAACGCGC GGGGAGAGGC

7681 GGTTTGCGTA TTGGGCGCTC TTCCGCTTCC TCGCTCACTG ACTCGCTGCG CTCGGTCGTT

7741 CGGCTGCGGC GAGCGGTATC AGCTCACTCA AAGGCGGTAA TACGGTTATC CACAGAATCA

7801 GGGGATAACG CAGGAAAGAA CATGTGAGCA AAAGGCCAGC AAAAGGCCAG GAACCGTAAA

7861 AAGGCCGCGT TGCTGGCGTT TTTCCATAGG CTCCGCCCCC CTGACGAGCA TCACAAAAAT

7921 CGACGCTCAA GTCAGAGGTG GCGAAACCCG ACAGGACTAT AAAGATACCA GGCGTTTCCC

7981 CCTGGAAGCT CCCTCGTGCG CTCTCCTGTT CCGACCCTGC CGCTTACCGG ATACCTGTCC

8041 GCCTTTCTCC CTTCGGGAAG CGTGGCGCTT TCTCATAGCT CACGCTGTAG GTATCTCAGT

8101 TCGGTGTAGG TCGTTCGCTC CAAGCTGGGC TGTGTGCACG AACCCCCCGT TCAGCCCGAC

8161 CGCTGCGCCT TATCCGGTAA CTATCGTCTT GAGTCCAACC CGGTAAGACA CGACTTATCG

8221 CCACTGGCAG CAGCCACTGG TAACAGGATT AGCAGAGCGA GGTATGTAGG CGGTGCTACA

8281 GAGTTCTTGA AGTGGTGGCC TAACTACGGC TACACTAGAA GGACAGTATT TGGTATCTGC

8341 GCTCTGCTGA AGCCAGTTAC CTTCGGAAAA AGAGTTGGTA GCTCTTGATC CGGCAAACAA

8401 ACCACCGCTG GTAGCGGTGG TTTTTTTGTT TGCAAGCAGC AGATTACGCG CAGAAAAAAA

8461 GGATCTCAAG AAGATCCTTT GATCTTTTCT ACGGGGTCTG ACGCTCAGTG GAACGAAAAC

8521 TCACGTTAAG GGATTTTGGT CATGAGATTA TCAAAAAGGA TCTTCACCTA GATCCTTTTA

8581 AATTAAAAAT GAAGTTTTAA ATCAATCTAA AGTATATATG AGTAAACTTG GTCTGACAGT

8641 TACCAATGCT TAATCAGTGA GGCACCTATC TCAGCGATCT GTCTATTTCG TTCATCCATA

8701 GTTGCCTGAC TCCCCGTCGT GTAGATAACT ACGATACGGG AGGGCTTACC ATCTGGCCCC

8761 AGTGCTGCAA TGATACCGCG AGACCCACGC TCACCGGCTC CAGATTTATC AGCAATAAAC

8821 CAGCCAGCCG GAAGGGCCGA GCGCAGAAGT GGTCCTGCAA CTTTATCCGC CTCCATCCAG

8881 TCTATTAATT GTTGCCGGGA AGCTAGAGTA AGTAGTTCGC CAGTTAATAG TTTGCGCAAC

8941 GTTGTTGCCA TTGCTACAGG CATCGTGGTG TCACGCTCGT CGTTTGGTAT GGCTTCATTC

9001 AGCTCCGGTT CCCAACGATC AAGGCGAGTT ACATGATCCC CCATGTTGTG CAAAAAAGCG

9061 GTTAGCTCCT TCGGTCCTCC GATCGTTGTC AGAAGTAAGT TGGCCGCAGT GTTATCACTC

9121 ATGGTTATGG CAGCACTGCA TAATTCTCTT ACTGTCATGC CATCCGTAAG ATGCTTTTCT

9181 GTGACTGGTG AGTACTCAAC CAAGTCATTC TGAGAATAGT GTATGCGGCG ACCGAGTTGC

9241 TCTTGCCCGG CGTCAATACG GGATAATACC GCGCCACATA GCAGAACTTT AAAAGTGCTC

9301 ATCATTGGAA AACGTTCTTC GGGGCGAAAA CTCTCAAGGA TCTTACCGCT GTTGAGATCC

9361 AGTTCGATGT AACCCACTCG TGCACCCAAC TGATCTTCAG CATCTTTTAC TTTCACCAGC

9421 GTTTCTGGGT GAGCAAAAAC AGGAAGGCAA AATGCCGCAA AAAAGGGAAT AAGGGCGACA

9481 CGGAAATGTT GAATACTCAT ACTCTTCCTT TTTCAATATT ATTGAAGCAT TTATCAGGGT

9541 TATTGTCTCA TGAGCGGATA CATATTTGAA TGTATTTAGA AAAATAAACA AATAGGGGTT

9601 CCGCGCACAT TTCCCCGAAA AGTGCCAC

//
